# UV-polymerizable methacrylated gelatin (GelMA)-based hydrogel containing tannic acids for wound healing

**DOI:** 10.1007/s13346-023-01383-y

**Published:** 2023-07-20

**Authors:** Marismar F. do Nascimento, Clauberto R. de Oliveira, Juliana C. Cardoso, Natalia C. T. Bordignon, Rogério Gondak, Patrícia Severino, Eliana B. Souto, Ricardo L. C. de Albuquerque Júnior

**Affiliations:** 1 University of Pernambuco, Campus Petrolina, Petrolina, Pernambuco 56328-900 Brazil; 2 Biotechnological Postgraduate Program–RENORBIO, Federal University of Sergipe, São Cristóvão, Sergipe 49100-000 Brazil; 3 Postgraduate Program in Health and Environment, Tiradentes University, Aracaju, Sergipe 49032-490 Brazil; 4 Department of Dentistry, Post-Graduating Program in Dentistry, Federal University of Santa Catarina, Florianópolis, 88040-370 Brazil; 5 Department of Pathology, Health Sciences Center, Federal University of Santa Catarina, R. Delfino Conti, S/N, Florianópolis, Santa Catarina 88040-370 Brazil; 6 Post-Graduating Program in Industrial Biotechnology, University of Tiradentes, Av. Murilo Dantas, 300, Aracaju, 49010-390 Brazil; 7 UCIBIO–Applied Molecular Biosciences Unit, MEDTECH, Laboratory of Pharmaceutical Technology, Department of Drug Sciences, Faculty of Pharmacy, University of Porto, 4050-313 Porto, Portugal; 8 Associate Laboratory i4HB, Department of Pharmaceutical Technology, Faculty of Pharmacy, Institute for Health and Bioeconomy, University of Porto, Rua de Jorge Viterbo Ferreira, 228, 4050-313 Porto, Portugal

**Keywords:** *Punica granatum* aqueous extract, Gelatin methacryloyl hydrogel, GelMA, Iridoids, Wistar rats

## Abstract

Gelatin-based photopolymerizable methacrylate hydrogel (GelMA) is a promising biomaterial for in situ drug delivery, while aqueous extract of *Punica granatum* (AEPG) peel fruit rich in gallic acid and ellagic acid is used to improve wound healing. The aim of this study was to develop and analyze the healing properties of GelMA containing AEPG, gallic acid, or ellagic acid in a rodent model. GelMA hydrogels containing 5% AEPG (GelMA-PG), 1.6% gallic acid (GelMA-GA), or 2.1% ellagic acid (GelMA-EA) were produced and their mechanical properties, enzymatic degradation, and thermogravimetric profile determined. Wound closure rates, healing histological grading, and immunohistochemical counts of myofibroblasts were assessed over time. The swelling of hydrogels varied between 50 and 90%, and GelMA exhibited a higher swelling than the other groups. The GPG samples showed higher compression and Young’s moduli than GelMA, GGA, and GAE. All samples degraded around 95% in 48 h. GPG and GGA significantly accelerated wound closure, improved collagenization, increased histological grading, and hastened myofibroblast differentiation in comparison to the control, GelMA, and GEA. GelMA containing AEPG (GPG) improved wound healing, and although gallic acid is the major responsible for such biological activity, a potential synergic effect played by other polyphenols present in the extract is evident.

**Graphical Abstract:**

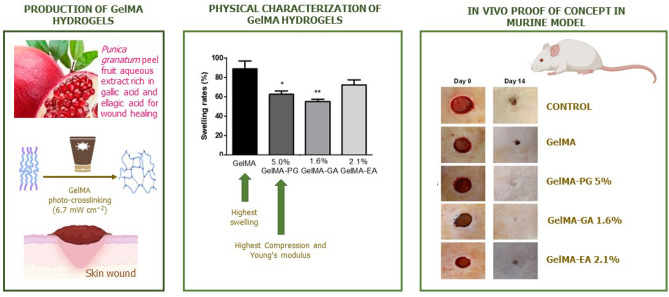

## Introduction

The skin integrity can be compromised by chronic diseases, such as diabetes and vascular diseases, but also by physical injuries (e.g., accidents and falls, high pressure, surgical interventions) and body immobility. All these factors compromise the role of the skin in keeping homeostasis, increasing the risk of infection, and may even result in high morbidity rates [1].

Wound dressings are instrumental in standard wound treatments, since these delivery systems have the capacity to physically protect the wound against mechanical injury, absorb its drainage, and provide the required moisture to foster and optimize re-epithelialization [2]. Several biomaterials with physiological and mechanical properties that resemble epidermal and dermal tissues have been proposed for the design of optimal wound dressings [3–5], aiming to accelerate wound healing and reconstitution of injured skin [6]. However, the design of a reliable and cost-effective wound dressing is still a challenging task, provided that it should respond to the wound drainage and its depth [7].

Among potential biomaterials, hydrogels are reported to be useful to create the required moist environment in which dermal cells (e.g., fibroblasts, macrophages, stem cells, adipocytes, mast and Schwann cells) can thrive for further healing, while being comfortable to the patient. Hydrogels may also facilitate tissue regeneration by acting as a scaffold for stem cells and as a supplier of drugs and other biomolecules [8].

Hydrogels based on methacrylated gelatin (GelMA) obtained by ultraviolet (UV) photoreticulation have been reported as an excellent biomaterial for wound dressings because they allow for proper cell adhesion, are highly permeable to nutrients, proteins, and oxygen, and can be easily degraded by enzymes [9, 10]. GelMA is also reported to be an effective biopolymer to load and deliver bioactive compounds and other drugs to improve wound healing [11, 12].

The formulation of bioactive compounds, from isolated molecules to more complex substances (such as plant extracts), into collagen-based biomaterials (including hydrogels) for wound dressing, has been largely used as a strategy to improve the healing properties of hydrogels [13]. These functional hydrogels are designed, not only to protect the wound bed and promote a proper environment for cell adhesion and proliferation but also to deliver substances that play important pathophysiological functions over the time-course of wound healing (e.g., with antimicrobial, anti-inflammatory, and anti-oxidation activities) and contribute to hemostasis [14, 15].

The pomegranate (*Punica granatum* Linn) fruit is known for its composition rich in phenolics and tannins [16]. The aqueous extracts of pomegranate peel have been described to stimulate collagen synthesis and proliferation of dermal fibroblasts, besides the potential to impair the activity of major collagen-degrading enzymes [17].

Based on documented evidence that *Punica granatum* extracts improve wound healing, both in vitro and in vivo [18–20], we have recently developed gelatin-based membranes containing aqueous extracts of the fruit peel and evaluated their potential to promote major histological changes towards wound healing [21], namely, improved deposition and arrangement of collagen fibers, formation of new granular tissue, and development of cutaneous appendages.

The biological activities of the aqueous extract of *Punica granatum* are attributed to its rich composition in phenolic compounds, particularly tannins, such as gallic and ellagic acid [22]. Ellagic acid is a well-known phytochemical metabolite that shows anti-inflammatory activity by changing pro-inflammatory mediators (tumor necrosis factor-α, interleukin-1β, interleukin-6) and by reducing the activity of nuclear factor-κB while increasing nuclear factor erythroid 2-related factor 2 expression [23]. Gallic acid is also known for its anti-inflammatory properties and for being able to inhibit keloid-derived fibroblast proliferation and migration, by downregulating matrix metalloproteinase-1 and -3 while upregulates tissue inhibitors of metalloproteinase-1, by suppressing the AKT/ERK signaling pathway [24]. Both polyphenols, ellagic and gallic acids, are potentially capable of modulating the key phases of the wound healing process, such as inflammation, angiogenesis, and collagenization [21].

Although the functionalization of methacrylated gelatin (GelMA) with tannic compounds, such as gallic acid, has been previously reported [25], there are still no studies either addressing the functionalization with ellagic acid and/or evaluating the healing activity of these functionalized photopolymerizable hydrogels in in vivo models. Therefore, the aim of this study was to design, characterize, and evaluate the healing potential of UV-polymerizable hydrogels based on methacrylated gelatin (GelMA) containing *Punica granatum* Linn peel extract and polyphenolic compounds for use as dressings on open wounds.

## Material and methods

### Plant extract material

The aqueous extract of pomegranate (*Punica granatum* Linn) fruit peel containing 32.24 mg/g of gallic acid and 41.67 mg/g of ellagic acid was prepared and characterized, as previously described by do Nascimento et al. [21]. Briefly, the peels of *P. granatum* were submitted to a drying procedure at 55 °C for 5 days. Then, 5 g of dried and powdered peels (32 mesh) was subjected to extraction by dynamic maceration in water (1:100 w/v) at 100 °C for 2 h, using a magnetic stirrer. The extract was filtered and concentrated in an airflow oven at 55 °C for 3 days.

### Production of gelatin methacryloyl prepolymer (GelMA)

For the production of prepolymer, a well-established, previously described protocol was followed [9, 26, 27]. Briefly, gelatin (10% w/v, Np Comercio De Produtos Alimenticios Ltda, São Paulo, Brazil) was dissolved in Dulbecco’s phosphate-buffered saline (DPBS; GIBCO) at 60 °C, pH 7.4, and methacrylic anhydride (1% v/v) was added to the solution (50 °C). After 3 h, the mixture was dialyzed against distilled water for 1 week. The material was subjected to lyophilization (− 55 °C and vacuum pump at 0.03–0.2 mbar) for 5 consecutive days using a Liotop L101 (Liobras, São Carlos, São Paulo, Brazil) to form the GelMA prepolymer. Subsequently, the photoinitiator Irgacure 2959 (2-hydroxyl-1-[4-(2-hydroxyethoxy) phenyl] -2-methyl-1-propanone, 0.5 w/v%, Sigma-Aldrich, St. Louis, MO, USA) was mixed to a GelMA prepolymer solution (10% w/v in DPBS, 80 °C). Photocrosslinking was achieved by exposing the solution to 6.7 mW cm^−2^ UV light Philips TL40W/12 RS (light intensity of 8.6 × 10^4^/cm^2^) (Koninklijke Philips N.V., Amsterdam, The Netherlands) for 60 s. The same formulation was prepared by adding AEPG (0.005 g), gallic acid (0.0016 g, > 99%, 3C_6_H_2_CO_2_H. H_2_O, MW: 188.13 g/mol, Sigma-Aldrich, St. Louis, MO, USA), or ellagic acid (0.0021 g, > 95%, C_14_H_6_O_8_, MW: 302.19 g/mol, Sigma-Aldrich) based on previously performed chemical analysis of the crude aqueous extract of *Punica granatum* (do Nascimento et al. [21]), to obtain the formulations composed of 5% GelMA-GPG, 1.6% GelMA-GA, and 2.1% GelMA-EA, respectively.

### Characterization of the GelMA-based formulations

#### Assessment of the swelling ratio (%)

The swelling ratio was assessed as described by Xiao et al. [28]. Briefly, 250 µL of each formulation was pipetted into a 0.75-mm diameter circular mold (*n* = 8) and exposed to UVB light (Philips TL40W/12 RS lamp at a distance of 4 cm above the irradiated area, light intensity of 8.6 × 10^4^/cm^2^ for 60 s, at room temperature). Then, 300 µL of PBS was added to the molds and incubated at room temperature (24 h). Subsequently, the weight was recorded, and the swelling ratio (SR%) was calculated according to the following equation:

$$\mathrm{SR}(\%)=\frac{\mathrm{fW}-\mathrm{iW}}{\mathrm{iW}}\times 100$$

where iW is the initial weight and fW is the final weight.

#### Analysis of mechanical properties

The mechanical properties of the photoactivated methacrylate hydrogel samples were assessed using a texturometer equipment (TA.XT2 Texture Analyzer, Stable Micro Systems, Surrey, UK), equipped with a 5-kg load cell, in compression mode at test speed of 2 mm/s and return speed of 5 mm/s. Compression and Young’s moduli were determined with the slope of the linear region corresponding to 0 and 5% of strain, as previously described by Nichol et al. [29].

#### In vitro enzymatic degradation assay

The formulations (GPG, GGA, and GAE) were evaluated in vitro with aliquots of 1.5 mL of each formulation of the hydrogels under study with the addition of 2.5 U/mL of collagenase type II solution (Worthington Biochemical Corporation, Lakewood, NJ, USA) and incubated at 37 °C for 3, 6, 12, 24, 36, and 48 h. After each time point, the excess collagenase was removed, the remaining hydrogel was washed with PBS, and then all the liquid was removed, and the sample was frozen (–4 °C) and then lyophilized. The percentage of degradation was calculated by determining the ration between the dry weight and the weight of the remaining hydrogels. The experiment was performed in sextuplicate.

#### Thermogravimetric (TG) and derivative thermogravimetric (DTG) analysis

The TG/DTG curves were obtained in a thermobalance DTG-60H Simultaneous DTA-TG apparatus (Shimadzu, Kyoto, Japan), in the temperature range of 25–800 °C, using a platinum sample holder containing 7.8 mg of the sample under dynamic N_2_ atmosphere (flow rate of gas of 50 mL/min) and heating rate of 10 °C/min.

### In vivo biological wound healing assay

#### Ethics issues

For the present work, ethics approval for conducting animal experimentation was obtained from the Ethical Committee for Animal Experimentation of University of Tiradentes and issued by the National Animal Experiment Control Council (CONCEA-Brazil) (approval #030915).

#### Animals and experimental procedures

Ninety male *Wistar rats* (280 g ± 20 g) were placed in cages with shaving bedding, changed daily, and kept at a controlled temperature of 22 °C, in a light/dark cycle of 12 h, receiving water ad libitum and standard diet Labina^®^ (Purina, São Paulo, Brazil). The animals were anesthetized (0.1 mL/100 g of 1 mL of 50 mg ketamine and 20 mg xylazine, *i.p.*), and after their backs were shaved, the antisepsis was performed with 1% topical povidone-iodine. Then, standard circular dermo epidermal wounds were induced in each animal using a 8.0-mm diameter stainless punch scalpel. In the immediate postoperative period, the animals received a prophylactic dose of 10 mg/kg of diclofenac potassium (*i.m.*). Subsequently, the animals were assigned into five groups (*n* = 6): CTR (untreated), GelMA (wounds filled with photoactivated hydrogel with no active compounds), GPG (wounds filled with 5% GelMA-PG), GGA (wounds filled with 1.6% GelMA-GA), and GEA (wounds filled with 2.1% GelMA-EA). The volume of hydrogel inserted into the wounds was 50 µL, and the photopolymerization was performed as described in the “Assessment of the swelling ratio (%)” section. After 3, 7, and 14 days, six animals from each group were euthanized using a lethal dose of xylazine/ketamine (1:1, 20 mg/kg). The dermal injured areas were surgically removed (approximately 1.0 cm of margins) and fixed in a 10% formalin solution (pH 7.4) for 48 h.

#### Assessment of wound closure rates (WCR)

The wounds of all the animals were photographed on days 0 (immediately after surgery), 3, 7, and 14 after surgery using a digital camera (Cybershot Sony HX-300) fixed on a tripod placed 20 cm above the wound surface. The images were recorded as.tiff format and analyzed using the software ImageJ^®^ (National Institutes of Health, Bethesda, MD, USA) to obtain a value for the total wound area. The percentage of wound closure (WCR%) was determined applying the following equation:

$$\mathrm{WCR}(\%)=\frac{\mathrm{fA}-\mathrm{iA}}{\mathrm{iA}}\times 100$$

where iA is the initial wound area and fA is the final wound area. Daily clinical evaluation of the wounds was also performed, according to the presence of clinical signs of an atypical course of wound healing (e.g., edema, suppuration, hemorrhage, and hyperemia).

#### Histological procedures and pathological analysis of the specimens

Formalin-fixed specimens were dehydrated, cleared, and embedded in paraffin, according to routine histological processing techniques. A total of nine serial histological slides (5 µm thick) were obtained from each embedded specimen, three of them (slides 1, 4, and 7) stained in hematoxylin–eosin staining (HE) (routine staining) and three in Sirius red (slides 2, 5, and 8) for further assessment of histological grading of wound healing. HE-stained histological sections were also used to perform a descriptive analysis of the inflammatory infiltrate, granulation tissue, fibrous scar, and epithelization of the wounds.

#### Assessment of the histological grading of wound healing (HGWH)

A semiquantitative scoring system was used to perform the histological grading of wound healing, as previously described by Gupta and Kumar [30]. This system is based on an ordinal scale with six histological criteria, as shown in Table 1. Three histological fields (100 × magnification, 0.25 mm^2^) in each histological section (one from each edge and one from the center of the wounds) were selected and analyzed. The final healing score in each case was obtained by adding the scores of the individual criteria. The data obtained were expressed as median, interquartile range, and maximum and minimum values. All histological sections were examined by two expert examiners, who were blinded to the groups during all the histological analysis.

**Table 1:** Histological criteria for histological grading of wound healing in dermal wounds (*HE*, hematoxylin–eosin staining)

**Histological parameter**	**Scoring system**	**Microscopy (histochemical technique)**
Inflammatory infiltrate	Plenty-1, moderate-2, a few-3	Light microscopy (HE)
Amount of granulation tissue	Profound-1, moderate-2, scanty-3, absent-4	Light microscopy (HE)
Orientation of collagen fibers	Vertical-1, mixed-2, horizontal-3	Polarized light (Sirius red)
Pattern of collagenization	Reticular-1, mixed-2, fascicle-3	Polarized light (Sirius red)
Amount of early collagen (type III)	Profound-1, moderate-2, minimum-3, absent-4	Polarized light (Sirius red)
Amount of mature collagen (type I)	Profound-1, moderate-2, minimum-3	Polarized light (Sirius red)

#### Assessment of collagenization ratio (CR)

The analysis of collagen fibers was carried out in three histological sections of each animal stained in Sirius red and analyzed under polarized light. Collagen fibers were classified according to their birefringence pattern (type III with green birefringence and type I with yellow/red birefringence), morphological features (stretched/wavy, thin/thick, short/long), and architectural arrangement (reticular, parallel, or fascicle). Therefore, three histological fields (100 × magnification, 0.25 mm^2^) of each histological section were selected as described in the “Assessment of the histological grading of wound healing (HGWH)” section, photomicrographed, and recorded as.tiff file type format. The percentage of the area containing collagen fibers was determined using the method of color cluster segmentation using the ImageJ^®^ software (version for Windows 1.8.0_172), as described by Miot and Brianezi [31]. First, the images were transformed into gray scale to select the color cluster based on the shades of pixels. The density of the collagen was assessed estimating the density of shade in relation to the background color. Data were expressed as the mean percentage of dermal wound collagenization per histological field (0.25 mm^2^) (mean ± standard deviation). The examiner was always blinded to the experimental groups during the histological analysis.

#### Immunohistochemical assessment of myofibroblasts

For myofibroblast immunodetection, deparaffinized histological slides 3, 6, and 9 were subjected to endogenous peroxidase activity blockade with 3% hydrogen peroxide and methyl alcohol (10 min in a dark room). Subsequent antigen recovery was performed by moist heat under pressure in 10-mM citrate buffer/pH 6.0 solution. The histological sections were incubated with anti-α-SMA antibody (clone 1A4, Dako, 1:100) for 30 min. The secondary antibody (SABC (streptavidin–biotin complex), catalog number SA1022) was incubated at 37 ℃ for 30 min. The reaction was revealed by incubating the histological slides with diaminobenzidine (DAB, Ventana Medical Systems, Tucson, AZ, USA) in a dark room for 30 min. Counterstaining was performed with Meyer’s hematoxylin (Sigma-Aldrich, St. Louis, MO, USA). Two histological slides of myofibroma were used as a positive control, whereas the negative control was obtained using two other slides from the same tumor, replacing the primary antibody by TRIS–HCl. The number of α-SMA-positive cells was counted in ten field histological series of each histological Sect. (400 × , analytical area corresponding to 0.025 mm^2^). Final data were expressed as mean ± standard deviation (SD) cells/field.

### Statistical analysis

All the data sets were subjected to analysis of normality distribution using Shapiro–Wilk’s test and homoscedasticity using Bartlett’s test. Data sets with symmetric (Gaussian) distribution and homoscedasticity were expressed as mean ± standard deviation (SD), and differences between means were analyzed using the two-way analysis of variance (ANOVA) followed by Tukey’s multiple comparison test. Data sets with asymmetric (non-Gaussian) distribution were expressed as median and interquartile range, and differences between medians were analyzed using the Kruskal–Wallis test followed by Dunn’s multiple comparison test. A significance level of 5% was adopted in all statistical tests applied in this study. The statistical analysis was carried out using the PRISM 7.0 software (GraphPad Software: La Jolla, CA, USA).

## Results

### Characterization of the hydrogels

**Fig. 1:**
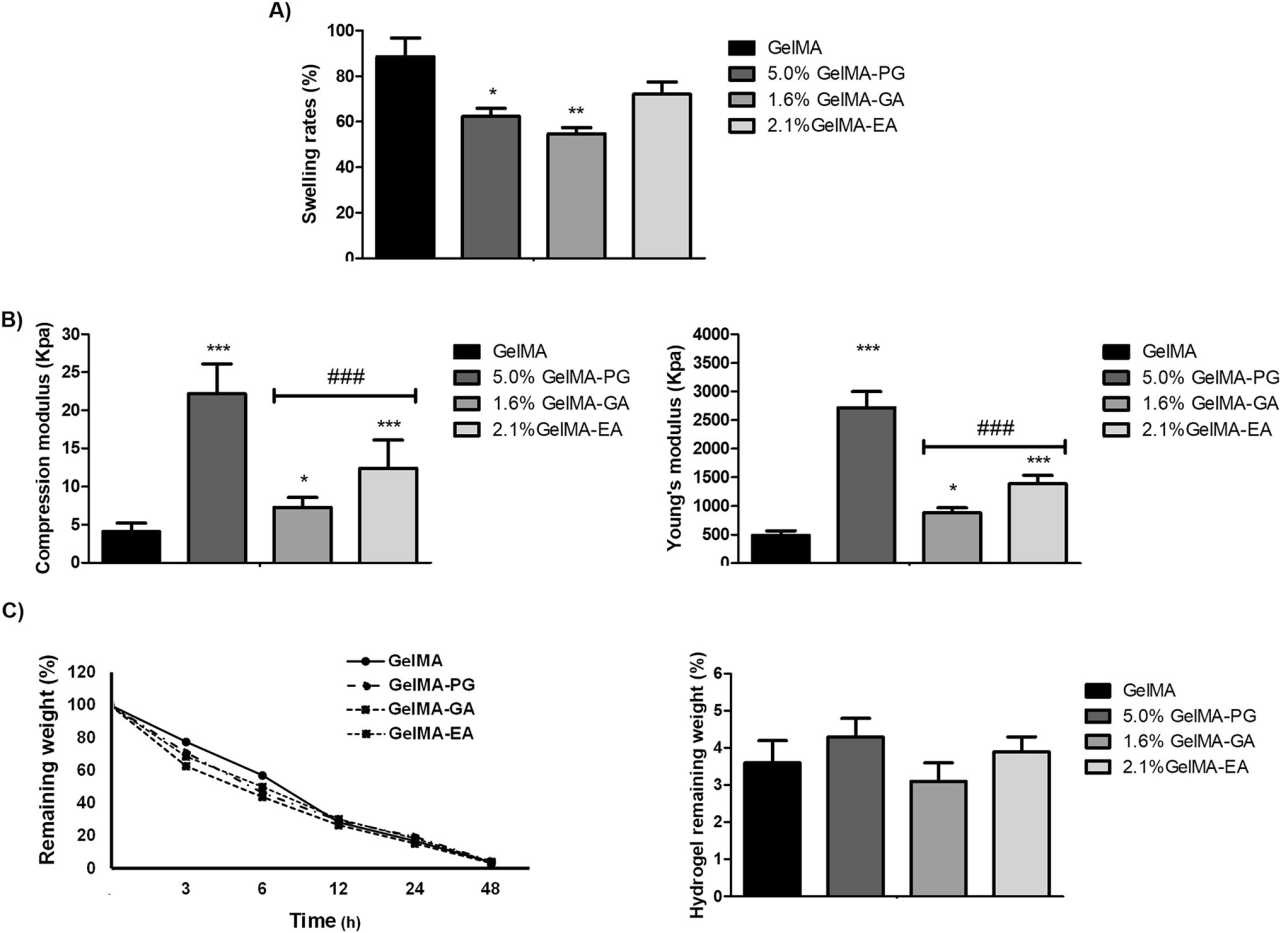
**A** Assessment of swelling rate, **B** compression and Young’s modulus, and **C** degradability rate and final weight of the hydrogels. Data are expressed as mean ± SD (*n* = 8). Significant differences in comparison to GelMA are expressed as *p < 0.05, **p < 0.01, and ***p < 0.001; significant differences in comparison to GelMA-PG are expressed as.^###^*p* < 0.001 (ANOVA and Tukey’s multiple comparison test)

Data on the characterization of the hydrogels regarding the swelling rates (SR), mechanical properties, and enzymatic degradation are shown in Fig. 1. The SR hydrogels ranged from 50 to 90% (Fig. 1A). The SR of GelMA (88.6 ± 8.3%) was significantly greater than the formulations 5% GelMA-PG (61.4 ± 3.6%; *p* < 0.05) and 1.6% GelMA-GA (54.7 ± 1.9%; *p* < 0.01), but no difference was observed in comparison to 2.1% GelMA-EA (72.1 ± 5.3%; *p* > 0.05). The analysis of the mechanical properties of the hydrogels (Fig. 1B) shows that the compression modulus (tension) and Young’s modulus (stiffness) of the formulations 5% GelMA-PG (22.2 ± 4.2 kPa), 1.6% GelMA-GA (7.3 ± 1.3 kPa), and 2.1% GelMA-EA (12.4 ± 3.7 kPa) were significantly greater than GelMA (4.1 ± 1.1 kPa) (*p* < 0.05). Furthermore, GelMA-PG exhibited greater compressive modulus and stiffness modulus compared to the GelMA-GA and GelMA-EA (*p* < 0.001), but there was no difference between the latter two samples (*p* > 0.05). All the formulations, including GelMA with no additional bioactive compound, exhibited a similar profile of enzymatic degradation characterized by progressive loss of weight over 48 h (Fig. 1C). At the end of the experimental period, the remaining weight of the hydrogel ranged approximately from 2.0 to 5.0%, and there was no significant difference between groups (*p* > 0.05).

**Fig. 2:**
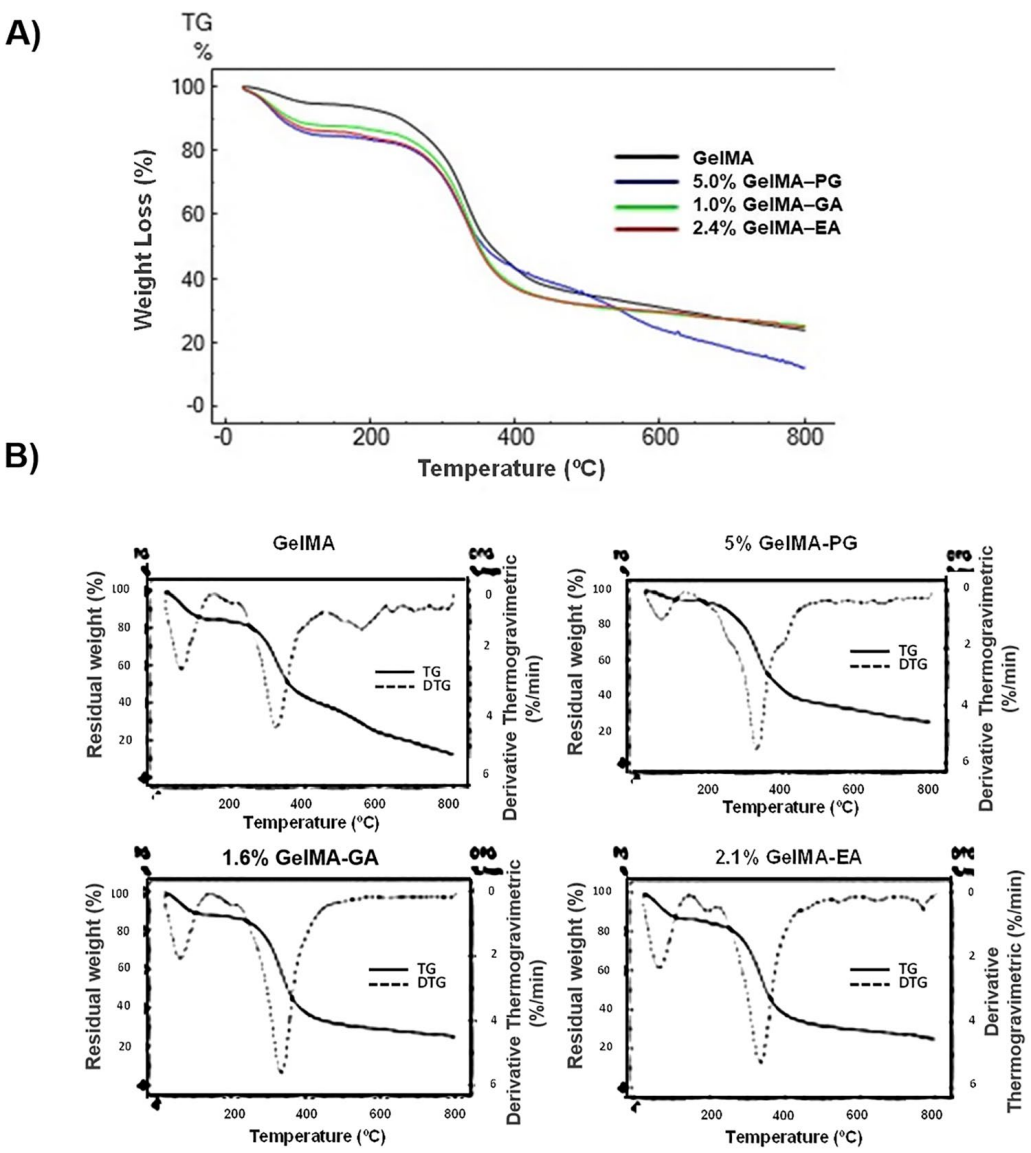
**A** Thermogravimetric and **B** derivative thermogravimetric curves of GelMA hydrogel without polyphenolic compounds (GelMA) and containing aqueous extract of *Punica granatum* (GelMA-PG), gallic acid (GelMA-GA), and ellagic acid (GelMA-EA) and in scan up to 800 °C

Thermal characterization is shown in Fig. 2. TG/DTG thermograms revealed that all samples exhibited three main weight loss events. In the first event, two stages of water loss were observed, one between 90 and 110 °C, possibly related to the loss of water adsorbed on the surface of the hydrogels, and the other between 110 and 230 °C, potentially associated with structural water loss. In the second event, the weight loss occurred between 230 and 360 °C and was attributed to the degradation of the gelatin organic matter. From 600 °C (third event) onwards, the weight loss was attributed to loss of remaining organic matter and impurities (e.g., inorganic matter).

### Effect of the photopolymerizable hydrogels on wound closure rates

For all of the groups (Fig. 2A), the wound closure rate was found to be progressive, centripetal, and without signs of clinical changes suggestive of impaired repair, such as abscess formation, edema, hyperemia, or hemorrhage. The mean WCR of the groups is shown in Fig. 2B. On day 3, the mean WCR values of the groups—GelMA (19.5 ± 4.0%), GelMA-PG (23.4 ± 4.6%), (GelMA-GA (20.8 ± 5.5%), and GelMA-EA (23.9 ± 6.1%)—were all significantly greater than that of CTR (10.8 ± 3.4%) (*p* < 0.01). Notwithstanding, no significant difference was observed between the groups treated with the different photopolymerizable hydrogels (*p* > 0.05). On days 7 and 14, the mean WCR values of GelMA-PG (42.0 ± 5.0% and 79.1 ± 9.2%) and GelMA-GA (38.7 ± 4.8% and 79.8 ± 4.6%) were significantly greater than those obtained for CTR (22.0 ± 3.4% and 63.9 ± 6.6%), GelMA (26.5 ± 3.3% and 69.3 ± 4.5%), and GelMA-EA (27.1 ± 6.6% and 63.1 ± 9.2%) (*p* < 0.001 and *p* < 0.05, respectively). However, there was no significant difference between those latter three groups, neither on day 7 nor on day 14 (*p* > 0.05).

### Effect of the photopolymerizable hydrogels on the pathological features and histological grading of wound healing

**Fig. 3:**
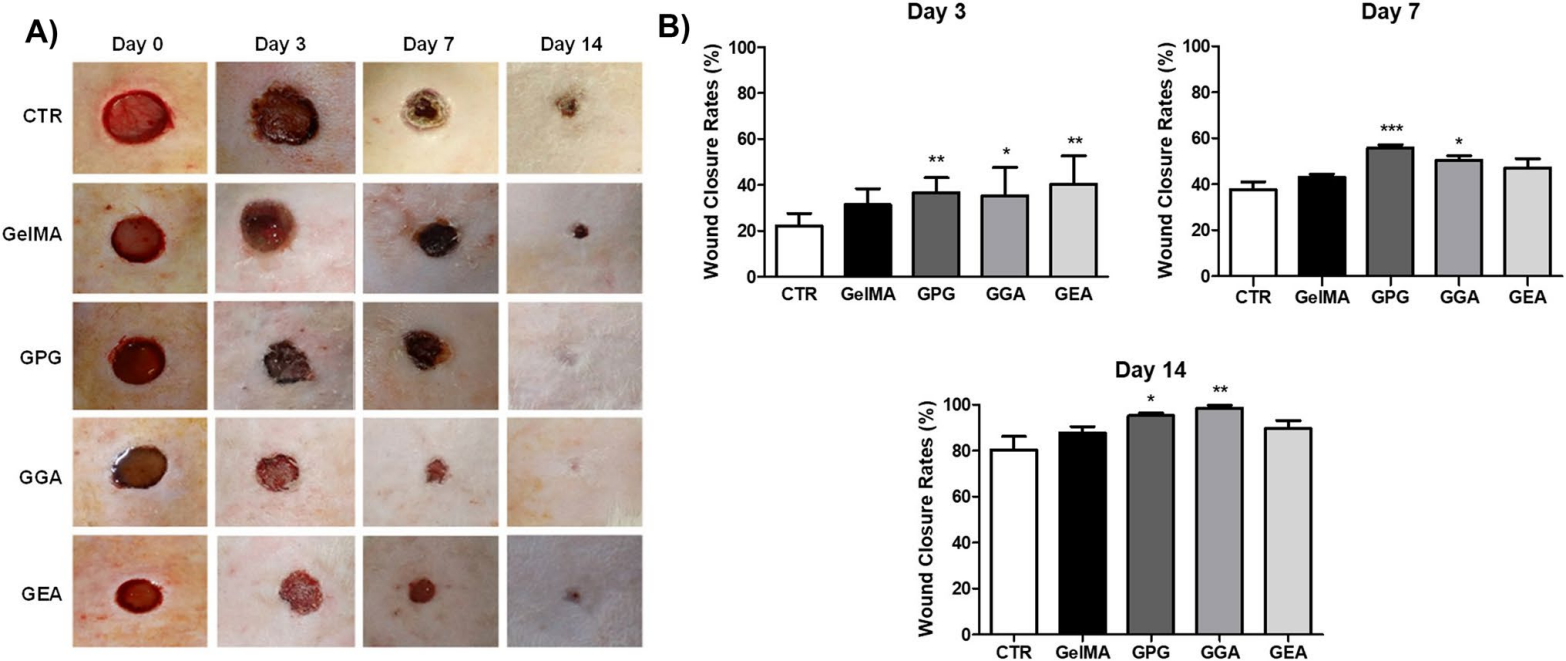
**A** Digital photographs of skin wounds in the CTR (control group with unfilled wounds), GelMA (wounds filled with GelMA hydrogel), GPG (wounds filled with GelMA hydrogel containing aqueous extract of *Punica granatum*), GGA (wounds filled with hydrogel GelMA containing gallic acid), and GEA (wounds filled with GelMA hydrogel containing ellagic acid) and **B** assessment of wound closure rates over the time-course of wound healing. Data are expressed as mean ± SD. Significant difference compared to CTR are expressed as ***p < 0.001, **p < 0.01, and *p < 0.05 (ANOVA and Tukey’s multiple comparison post-test, *n* = 6 animals/group)

Data about the pathological analysis and histological grading of wound healing over the time-course of the experiment are summarized in Fig. 3A and B. On day 3, the wounds were characterized by severe inflammatory infiltrate, predominantly composed of polymorphonuclear neutrophils, particularly at the top of the wounds, and serofibrinous edema of the connective tissue concentrated in the center and at the bottom of the wounds. The edema was more intense in CTR in comparison to the other groups. The histological grading of wound healing in GPG (9.00; 8.00–9.75), GGA (8.00; 7.00–9.00), and GEA (8.00; − 8.00 to 9.00) was significantly greater than in CTR (6.00; 6.00–7.00) (*p* < 0.001), but there was no significant difference between these groups and GelMA (7.00; 7.00–8.00) (*p* > 0.05). On day 7, the major pathological feature observed in all the groups was the formation of an exuberant granulation tissue filling the wounds. The granulation tissue was composed of many flattened, slit-shaped capillaries arranged perpendicular to the wound surface, supported by a delicate fibrillary collagen network of variable density. Re-epithelialization was ongoing, and it covered between 30 and 60% of the surface of the wounds.

The histological grading of wound healing in GelMA (11.00; 10.00–11.75) and GEA (12.00; 12.25–14.00) was significantly greater than in CTR (10.00; 9.00–11.75) (*p* < 0.05), whereas the grading in GPG (13.00; 12.00–15.00) and GGA (13.00; 12.00–15.00) was significantly greater than in all the other groups (*p* < 0.001).

On day 14, the specimens were represented by a primary fibrous scar, composed of parallel-arranged collagen fibers associated to spindle-shaped cells interpreted as fibroblasts or myofibroblasts. Residual granulation tissue was observed in all cases of CTR. Re-epithelization was advanced in all the cases (over 70%), and only GPG and GGA had 100% of the wound surfaces fully re-epithelialized. Small epithelial buddings compatible with rudimentary cutaneous appendages were seen in all groups but were more numerous in GPG and GGA. The groups GPG (16.00; 15.00–17.00) and GGA (16.00; 15.00–16.00) had greater histological grading of wound healing than the others (*p* < 0.05), whereas GelMA (15.00; 14.00–16.00) and GEA (15.00; 13.00–15.00) were significantly greater than CTR (13.00; 12.00–14.00) (*p* < 0.05).

### Effect of the photopolymerizable hydrogels on the wound collagenization

**Fig. 4:**
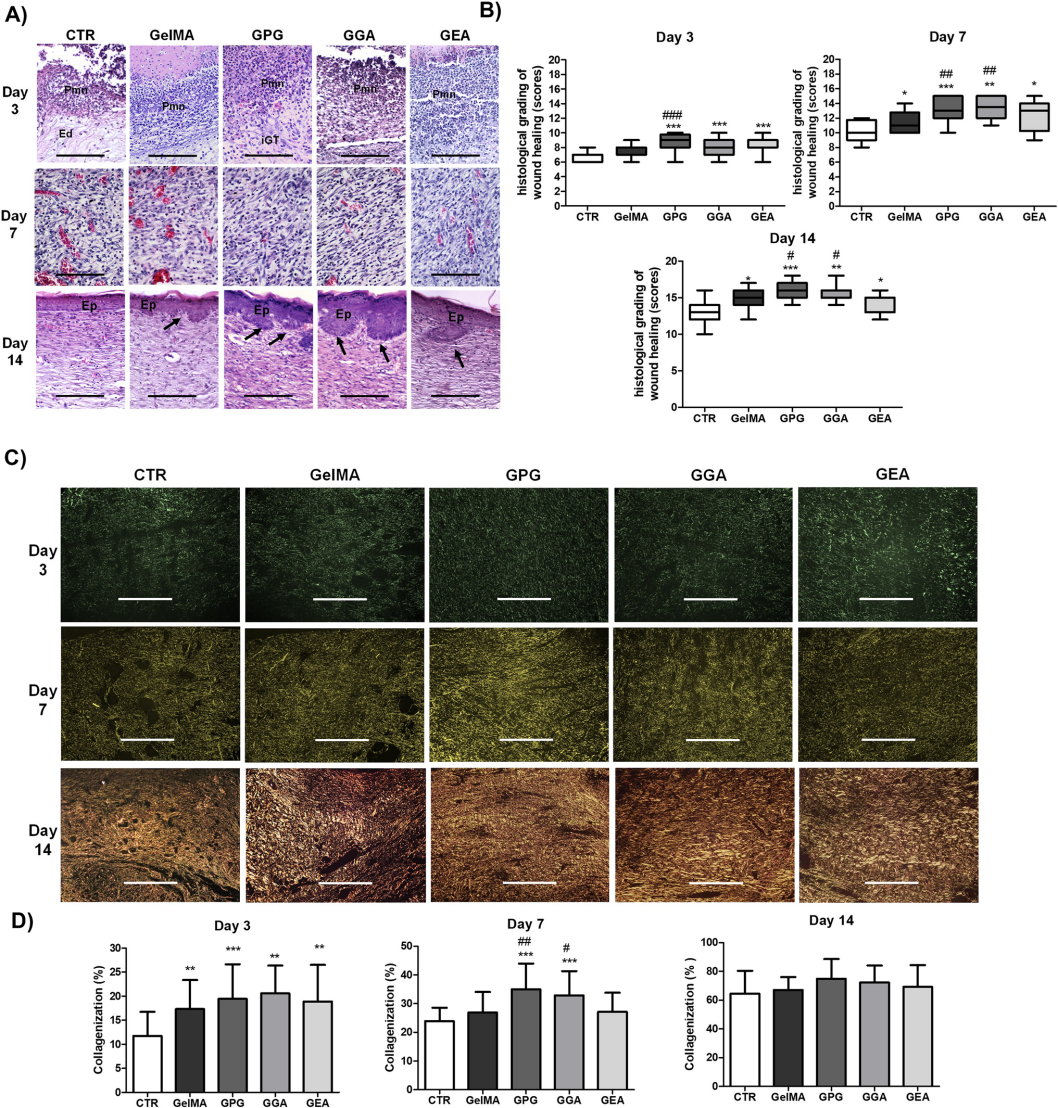
**A** Hematoxylin–eosin-stained histological slides (400 ×) showing the pathological features observed in the experimental groups over the wound healing time-course characterized by acute inflammation, rich in polymorphonuclear neutrophils (pmn), on day 3, exuberant granulation tissue on day 7, and primary fibrous scar on day 14. Re-epithelization of the wound surfaces (Ep) and the presence of epithelial biddings compatible with rudimentary cutaneous appendages (arrows). **B** Assessment of histological grading of wound healing over the time-course of the experiment (data expressed as median; interquartile range, *n* = 6 animals/group and 3 histological slides/animal). **C** Sirius red-stained histological slides showing the pattern of collagen deposition over the experimental time (polarized light, 100 ×). The birefringence is green for type I collagen and yellow-gold for type I collagen. **D** Assessment of the mean percentage of collagenization in the wounds over the experimental time (data expressed as mean ± SD, *n* = 6 animals/group and 3 histological slides/animal). Comparisons between groups of the scores of histological grading of wound healing were performed with Kruskal–Wallis’ test and Dunn’s multiple comparison test), whereas comparisons of the mean percentage of collagenization were performed using ANOVA and Tukey’s multiple comparison test). Significant differences compared to CTR are expressed as *p < 0.05, **p < 0.01, and ***p < 0.001; significant differences compared to GelMA and GEA are expressed as ^#^*p* < 0.05, ^##^*p* < 0.01, and ^###^*p* < 0.001. Black bar: 250 µm; white bar: 500 µm

Data about the pathological analysis and assessment of the mean percentage of collagenization of wound healing over the time-course of the experiment are shown in Fig. 4A and B. On day 3, collagenization was mild and represented by thin, short, and delicate collagen fibrils exhibiting green birefringence (type III collagen) and irregular arrangement. The interfibrillar spaces were large and abundant. The mean percentage of collagenization in GelMA (17.3 ± 6.0%), GPG (19.4 ± 7.2%), GGA (20.5 ± 5.7%), and GEA (18.9 ± 7.6%) was significantly greater than in CTR (11.7 ± 5.1%) (*p* < 0.01), but there was no difference between the groups treated with hydrogels (*p* > 0.05). On day 7, the green fibrils and fibers of type III collagen were still predominant, but they were organized in a denser reticular arrangement. The interfibrillar spaces, although reduced, were still conspicuous. The mean percentage of collagenization in GPG (35.0 ± 8.9%) and GGA (32.9 ± 8.4%) was significantly greater than in CTR (23.9 ± 4.6%; *p* < 0.001), GelMA (26.8 ± 7.1%; *p* < 0.05), and GEA (27.1 ± 6.6%; *p* < 0.01). No significant difference was observed between CTR, GelMA, and GEA (*p* > 0.05). On day 14, most of the type III collagen fibrils were replaced by longer, thicker, and coarser type I collagen fibers, with yellow-gold birefringence, and organized in an arrangement predominantly parallel to the wound surface. There was a clear reduction in interfibrillar spaces in all the groups. However, no significant difference was observed in the mean percentage of collagenization of CTR (64.5 ± 15.8%), GelMA (67.1 ± 8.9%), GPG (74.8 ± 13.7%), GGA (72.3 ± 11.8%), and GEA (69.1 ± 15.2%) (*p* > 0.05).

### Effect of the photopolymerizable hydrogels on the myofibroblast differentiation

**Fig. 5:**
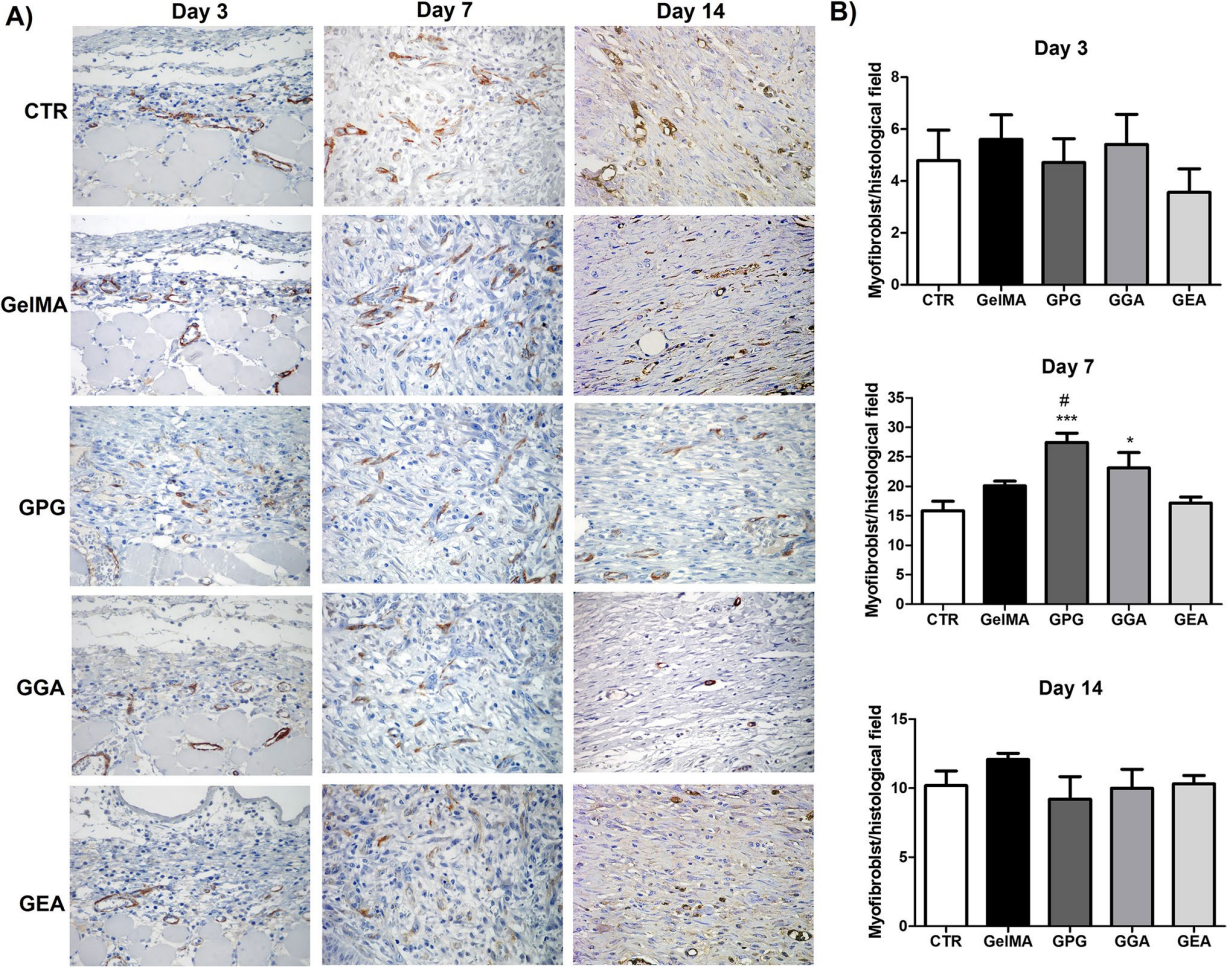
**A** Immunohistochemical detection of α-SMA-positive myofibroblast over the course of wound healing (SABC, 400 ×). Myofibroblasts are identified as spindle-shaped cells exhibiting the brown color of the cytoplasm, typically found along collagen fibrils and fibers. **B** Assessment of the mean number of myofibroblasts per histological field (0.025 mm^2^). Data are expressed as mean ± SD. Significant differences compared to CTR are expressed as *p < 0.05 and **p < 0.01; significant differences compared to GelMA and GEA are expressed as.^#^*p* < 0.05 (ANOVA and Tukey’s multiple comparison test, *n* = 6 animals/group and 3 histological slides/animal)

Myofibroblasts were identified through the cytoplasmic brown color as shown in Fig. 5A and B. On day 3, α-SMA-positive cells were mostly found surrounding small capillaries (cells interpreted as pericytes) at the bottom of the wounds, whereas myofibroblasts typically found along collagen fibrils were rare.

The mean number of myofibroblasts per histological field varied from 1.98 to 10.12, and no significant difference was observed between groups (*p* > 0.05). The amount of α-SMA-positive myofibroblasts increased significantly from days 3 to 7 in all the groups (*p* < 0.001), and the immunostained myofibroblasts were found adjacent to collagen fibers and fibrils, both parallel and perpendicular to the wound surface. However, the mean number of myofibroblasts was significantly greater in GPG (27.4 ± 4.1; *p* < 0.001) and GGA (23.1 ± 7.3; *p* < 0.05) than in CTR (15.8 ± 4.2). GPG was also significantly greater than GelMA (20.1 ± 2.1; *p* < 0.05) and GEA (17.1 ± 2.8; *p* < 0.05). No significant difference was found in any other comparison between groups at this experimental time (*p* > 0.05). In opposite to what occurred between days 3 and 7, the amount of myofibroblasts immunohistochemically detectable significantly decreased from days 7 to 14 in all the groups (*p* < 0.01). The number of myofibroblasts ranged from 4.3 to 15.4 cells per histological field, and no significant difference was observed between groups (*p* > 0.05). Interestingly, despite the decrease in the myofibroblast amount, the mean number of myofibroblasts on day 14 was still significantly greater than on day 3 in all the groups (*p* < 0.01).

## Discussion

In the present study, 10% GelMA prepolymer was used as the starting concentration for polymeric formulations containing bioactive compounds for wound filling. GelMA prepared at 10% was chosen because it does not undergo permanent deformations when subjected to up to 50-N load [32], favors cell adhesion and migration, and allows easy enzymatic degradation [9, 10]. As summarized in Table 2, a range of hydrogels containing ellagitannins for wound healing applications has been reported in the last 3 years, most of them containing gallic acid. Although many studies with hydrogels containing *P. granatum* extract and its major chemical compounds in in vivo models of healing have been previously performed, to the best of our knowledge, our study is the first to describe the use of a photopolymerizable gelatin-based polymer matrix as a delivery system for bioactive compounds. Compared to previously reported conventional hydrogels, the GelMA used in the current study provides better adaptability and adhesion to the wound bed and greater mechanical strength [9], besides its well-known and well-established cytocompatibility profile [33–37].

**Table 2:** Examples of biomaterial-based hydrogels containing tannic acids for wound healing applications tested in rodent models and published over the last 3 years

**Biomaterial**	**Rodent model**	**Major results**	**Reference**
Ellagic acid-cyclodextrin inclusion complex-loaded thiol-ene hydrogel	Infected wounds	Improvement of wound closure and promotion of promote angiogenesis and collagen deposition on histological basis	[44]
Hydrogel based on diammonium glycyrrhizinate and gallic acid	Infected wounds	Improvement of wound closure and denser connective tissues, better formation of dermal appendages (including hair follicles and sebaceous glands), and thicker and closer epidermal tissue on histological basis	[45]
Sodium alginate-based hydrogel with gallic acid-functionalized silver nanoparticles	Infected wounds	Improvement of wound closure and decrease in the expression of IL-6 and TNF-α to alleviate the inflammatory response and promotion of angiogenesis by upregulating CD31, α-SMA, and VEGF expressions	[46]
Fiber hydrogel constructed by gallic acid (GA) and phycocyanin (PC)	Both non-infected and infected wounds	Enabled adherence to the moist wound tissue and attenuation of inflammation and acceleration of wound healing both in normal mice and bacterium-infected mice through regulating the expression of the tight junction protein and the alleviation of oxidative stress	[47]
Double network hydrogels using agarose and gallic acid	Infected wounds	Improvement of wound closure and reduction of inflammation on histological analysis	[48]
Sustained-release hydrogel using gallic acid and lysozyme	Infected wounds	Improvement of wound closure by suppressing the expression of pro-inflammatory-related genes	[49]
Shape-adaptive gallic acid-driven multifunctional adhesive hydrogel loaded with scolopin2	Non-infected wounds	Improvement of wound closure in the first 7 days, complete regeneration of the skin appendages, and dermal and no residual inflammation on histological analysis and increased of CD31-positive vessel neoformation	[50]
Gallic acid-functionalized injectable hyaluronic acid hydrogel	Infected wounds	Improvement of wound closure and earlier recovery of cutaneous appendages, acceleration of the granulation tissue maturation, and most regular and intensive collagenization on histological basis	[51]
Self-assembly of gallic acid-constructed hydrogel	Infected wounds	Improvement of wound closure and reduction of the inflammatory response by mediation of inflammation signaling pathways	[52]
Resveratrol-triggered self-assembly of gallic acid-constructed hydrogel	Infected wounds	Improvement of wound closure and earlier completed epithelization and greater epidermal thickening on histological basis	[53]
Hexanoyl glycol chitosan with gallic acid	Non-infected wounds	Improvement of wound closure and tissue regeneration by upregulating growth factors (TGF, EGF, and VEGF) and recruiting fibroblasts	[54]

A hydrogel for wound filling must be permeable and have the adequate swelling capacity [38]. We found that incorporating aqueous extract of *Punica granatum*, gallic acid, and ellagic acid in a GelMA-based hydrogel, decreased the swelling index and increased the mechanical resistance (tensile modulus and stiffness) of the biomaterial.

The swelling index of the gels depends on the hydrogen bonds between the polymer and the solvent [29]. Thus, the ideal swelling of hydrogels occurs when there is a balance between the osmotic driving forces, allowing the entry of water or biological fluids into the hydrophilic matrix, and the cohesive forces exerted by the polymeric plot [39]. These cohesive forces resist the expansion of the hydrogel, preventing the polymers from dissolving in water. The extent of such forces particularly depends on the crosslinking density of the hydrogel, in such a way that the more hydrophilic the polymer is, the greater the amount of total water absorbed; likewise, the greater the degree of reticulation, the lesser its swelling. Thus, the reduction in the degree of swelling observed in active-loaded hydrogels (aqueous extract of *P. granatum*, ellagic acid, and gallic acid) could be the result of the connection of these compounds to the polar groups of the hydrogel, reducing its hydrophilicity. Furthermore, the addition of these active compounds may have promoted the filling of the pores of the polymeric matrix, impairing the diffusion of water molecules inside the hydrogel matrix and, thus, decreasing the swelling index [40]. Although swelling is a desired property in biomaterials for use in wounds, its reduction was considered a beneficial feature of the biomaterial developed in our study. This is because filling materials, such as GelMA hydrogels, are applied inside the injured area where loss of substance was seen. Thus, the exacerbated swelling of the biomaterial could cause compression of the wound edges and increase the magnitude of the local inflammatory response [41].

The incorporation of polyphenolic active compounds in the GelMA hydrogel also determined an increase in the compressive strength and Young’s modulus of the material. Similar results were recently reported after the incorporation of active compounds to GelMA [42]. These data are important because a filling biomaterial must be rigid enough to allow adhesion and sustain cell growth, or remain in place until absorbed, to promote proper tissue regeneration [43].

Another important parameter to support the choice of tissue substitutes is their biodegradability. Thus, in vitro assays of enzymatic degradation of wound filling biopolymers were run to understand the behavior of these biomaterials when in contact with biological constituents present in injured tissues, such as metalloproteinases (including collagenase) released by neutrophils and macrophages [55]. One of the most used enzymes for this purpose is type II collagenase, not only because it represents one of the most important in vivo extracellular matrix degradation enzymes but also because of its specificity to degrade gelatin-based products [28, 56].

In this study, hydrogels composed of methacrylated gelatin (GelMA 10%) showed high mass loss by enzymatic degradation (95% on average) in the first 48 h. After 48 h, the complete release of the bioactive components in the wound occurs. It is precisely the ideal time to control the inflammatory process and stimulate the phenomena of cell differentiation required for the formation of granulation tissue, rich in blood vessels and fibroblasts. The rat healing model assumes complete formation of granulation tissue in approximately 7 days. Therefore, the additional delivery of angiogenesis and collagenization modulating substances could promote important and undesired alterations in the course of healing. The main ones would be the formation of “spongy scar tissue” (excessive granulation tissue) or hypertrophic scar (excessive fibroblast activity). Similar results regarding GelMA degradation using the same sample incubation time and collagenase type II concentration were reported in previous studies [9, 32, 57, 58].

The weight of the hydrogels before (not dry, but after photopolymerization) and after incubation with collagenase was recorded. Despite the weight increase of the hydrogel with the swelling of the material, in the presence of collagenase, a weight loss was observed. After the degradation step, the mass loss was greater than the swelling gain, and it was thus possible to notice the degradability of the structure. Although the degradability profile of a biopolymer can be influenced by the incorporation of active chemical compounds, chemical structure and composition of the polymer, physicochemical factors (ionic charge, ionic strength, crosslinking, and pH), morphological criteria (amorphous, semicrystalline or crystalline, microstructure), route of administration, and by the site of action [59], the incorporation of phenolic compounds did not alter the degradation pattern of the hydrogels. Hydrogels are designed to degrade in tissue after implantation at a rate similar to the onset of granulation tissue formation [60]. Therefore, these results support the applicability of GelMA both as a filling material like a scaffold [61] and as delivery system for the release of active compounds at the wound site.

Thermogravimetric analysis was thus used to determine the polymer content as a function of temperature ramping with enhanced accuracy, being a fast, less expensive, and useful alternative over other analytical techniques. Although destructive, TGA is a high-throughput approach. Samples were heated up to 800 °C (Fig. 2), and, for all developed hydrogels, the highest residual weight was recorded between 200 and 500 °C. Up to 200 °C, the percentage of water loss was lower than 20%, which translates appropriate thermal stability of the developed hydrogels. The first stage of water loss was recorded between 90 and 110 °C attributed to the loss of water adsorbed on the surface of the hydrogels, whereas the most significant was recorded between 110 and 230 °C attributed to the loss of structural water. The presence of the aqueous extract of *Punica granatum* (GelMA-PG), gallic acid (GelMA-GA), and ellagic acid (GelMA-EA) did not influence the residual weight recorded up to 200 °C, when compared to plain GelMA (control).

Filling the wound area with GelMA containing AEPG (GPG), gallic acid (GGA), and ellagic acid (GEA) improved wound closure on day 3, likely in response to the anti-inflammatory activity of those phenolic compounds, also present in the extract [62]. Hence, the reduction of the area of the wounds filled with GPG, GGA, and GEA in the earlier stages of wound healing would have been a result of the lower severity of inflammation and interstitial edema, rather than a myofibroblast-related wound closure effect. On the 7th and 14th days, GPG and GGA showed significant improvement of wound closure. However, as GelMA and GEA did not show similar results, it is assumed that gallic acid might be the key molecule promoting wound closure acceleration. Supporting our findings, many other studies have previously demonstrated the gallic acid-related improvement of wound closure in wound healing assays [45, 54, 63–65]. The healing activity of the hydrogels containing gallic acid reported in the last 3 years (Table 2) has been related to the antioxidant and anti-inflammatory properties of this chemical compound. However, some studies have pointed at a potential modulatory effect of these ellagitannin-containing biomaterials on angiogenesis and collagenization steps of wound healing [44, 46, 50, 51, 54].

To clarify whether the wound closure acceleration was related to the improvement of the pathological features in the scar repairing, a wound healing histological grading system was applied in this study. The main advantages of the system used herein are as follows: (i) it encompasses the basic components of the healing process including angiogenesis, inflammation, fibroplasia, and epithelialization and differentiation and (ii) it is highly specific and standardized, while it is easily reproducible [30]. The very same system has already been successfully used in previous studies of wound healing in rodents [12, 21, 66]. The improvement of the histological grading of wound healing observed in all the groups whose skin wounds were filled with the gelatin-based biomaterials (GelMA, GPG, GGA, and GEA), in comparison to unfilled wounds (CTR), is possibly related to the bioconductive properties of the hydrogel, whose 3D-polymeric structure works as a scaffold for cell adhesion, proliferation, and migration, thus facilitating the earlier formation of granulation tissue and further reconstitution of the dermal lost tissue [9–12]. However, since GPG and GGA presented significantly better histological gradings over the time-course of the experiment, gallic acid released into the wound bed could likely be triggering additional pathophysiological, biochemical, or molecular scarring mechanisms, in addition to those promoted by GelMA, providing even better healing outcomes. Supporting these findings, gallic acid has previously been demonstrated to increase the viability of cultured cells subjected to oxidative stress through free radical scavenging, to modulate the expression of antioxidant genes in keratinocytes and fibroblasts, and to accelerate cell migration of keratinocytes and fibroblasts in an in vitro healing model [63]. In addition, it has been demonstrated that the healing properties of gallic acid-containing hydrogels could result from increased VEGF and TGF-β expressions, which would promote the improvement of the angiogenesis and myofibroblast differentiation, consequently improving the granulation tissue and collagen scar formation [46, 54].

Since the 2000s, the intensity of deposition, morphology, and architectural arrangement of collagen fibers are considered key events to assess the success of wound healing, particularly in secondary intention healing [67–69]. A significant increase in collagen deposition in wounds with great loss of substance can determine the formation of hypertrophic scars at the end of the process [70], whereas the reduced collagenization induces depressed/atrophic scarring [71]. The Sirius red histochemical technique, analyzed under polarized light microscopy, is highly sensitive and specific method for the identification and quantification of collagen fibers by means of birefringence patterns, differences in diameter, and structures of these fibers. Type III collagen forms thin fibers consisting of delicate fibrils, loosely arranged showing a weak greenish and yellow-green birefringence, whereas type I collagen forms fibers of variable thickness, arranged parallel or intertwined, with golden and reddish birefringence [72]. As observed in our study, the scarce deposition of type III collagen fibrils in the earliest stages of wound healing, which are further degraded and replaced by compact bundles (with less interfibrillar spaces) of type I collagen in the later stages, has already been reported in other studies [12, 21, 66]. This is because thin and delicate type III collagen fibrils are initially deposited in order to provide a three-dimensional scaffold that guides endothelial migration during the early stages of granulation tissue development. Subsequently, these fibers are progressively replaced by thicker type I collagen fibers with greater tensile strength as the scar tissue matures [73].

In a previous study, we have demonstrated that AEPG stimulates collagen deposition in rodent model of wound healing [21]. Hence, we investigated whether such a stimulatory effect would be promoted by gallic acid or ellagic acid found in AEPG. The application of GelMA improved type III collagen deposition on day three, regardless of whether or not it contains bioactive compounds, possibly by facilitating the influx of fibroblasts into the wound bed. However, GPG and GGA promoted greater improvement of collagenization on day seven, suggesting a possible role played by gallic acid on collagen synthesis during wound healing. The effects of gallic acid on collagen deposition are still controversial. Dermal fibroblasts treated with gallic acid-coated gold nanoparticles have been shown to prevent UV-induced photoaging by suppressing the expression of MMP-1, the major collagenase capable of destroying type I and III collagen [74]. In addition, gallic acid-induced upregulation of collagen synthesis required for proper wound healing has been reported and attributed to its antioxidant or anti-inflammatory activities [64]. On the other hand, other investigations pointed at a potential inhibitory effect of gallic acid on collagen synthesis in bleomycin-induced pulmonary fibrosis [75] and pressure overload-induced cardiac fibrosis [76]. This apparent paradox might be related to possible differences in the response pattern of dermal fibroblasts and those ones present in other connective tissues to gallic acid.

We have previously demonstrated histological evidence of the association between increased myofibroblast activity and improved wound healing promoted by the use of collagen-based membranes containing an aqueous extract of *Punica granatum* in rodent model [21]. Similarly, the AEPG delivered in GelMA also enhanced myofibroblast differentiation on day seven in the current study. The proliferative phase of wound healing that occurs from days seven to 14 in a rodent model is typically characterized by abundant myofibroblast differentiation [42], which explains the low myofibroblast number on day three. Moreover, the number of myofibroblasts was also increased in response to the delivery of gallic acid, but not ellagic acid, although the number of myofibroblast was even greater in wounds treated with AEPG than with gallic acid. These data suggest that gallic acid is at least in part responsible for the increased myofibroblast number on day 7, but other chemicals present in the extract might be playing a synergic or additional role on this pathophysiological event. Paradoxically, gallic acid has been demonstrated to induce apoptosis of excessive myofibroblasts via activation of p53 pathway, attenuating lung fibrosis [76]. Furthermore, GA treatments markedly suppressed the mRNA level of α-SMA, a local event needed to generate differentiated myofibroblasts [77]. Hence, although it is unlikely that gallic acid directly stimulated the formation of myofibroblasts, based on our results, a probable indirect effect on this pathophysiological event could still be assumed. In fact, as no significant difference in myofibroblast number was observed between groups on day 14, the greater number of myofibroblasts on day 7 could rather be a result of acceleration than the increase of myofibroblast differentiation. In this way, not only gallic acid but also other phenolic compounds are able to attenuate inflammation and oxidative stress in rodents [77, 78], which could ultimately favor the earlier development of an appropriate microenvironment for fibroblastic proliferation and subsequent myofibroblast differentiation. In addition, since myofibroblasts are specialized cells rich in contractile filaments of actin that facilitate rapid wound closure in healing tissues [79], it is possible to assume that the improvement in wound closure was mediated by earlier myofibroblast activity.

Interestingly, it is well established that ellagic acid is also able to modulate the inflammatory response and free radical formation [80, 81]. These data suggest that, even at lower concentrations, gallic acid was more efficient than ellagic acid to modulate the initial stages of wound healing. On the other hand, the presence of other phenolic compounds in the AEPG, such as ellagic acid [21], even in lower contractions, may have acted synergistically in modulating inflammation and inhibiting oxidative stress, which would have determined better results by using the GelMA containing the extract.

## Conclusions

In conclusion, the present study suggests that the photopolymerizable hydrogel based on methacrylated gelatin (GelMA) containing an aqueous extract of *Punica granatum* (AEPG) is a promising biomaterial to accelerate the healing of wounds with loss of substance. We demonstrated that this biomaterial not only has adequate mechanical properties for use as a moldable filling material for open wounds but also accelerates wound closure, improves the histological gradation of wound healing, and modulates myofibroblast differentiation in rodent model. Furthermore, our data suggest that gallic acid seems to represent the chemical compound present in the extract responsible for promoting the biological effects of this latter.

## Data Availability

The data sets generated during and/or analyzed during the current study are available from the corresponding author on reasonable request.
